# From Anatomical to Clinical DRLs: Establishing Indication-Based CT Dose Benchmarks in Saudi Arabia

**DOI:** 10.3390/diagnostics16121897

**Published:** 2026-06-18

**Authors:** Abir Bouaoun, Reem M. Althubaiti, Rudinah W. Edreess, Afnan A. Malaih

**Affiliations:** 1 Department of Radiologic Sciences, Faculty of Applied Medical Sciences, King Abdulaziz University, Jeddah 22252, Saudi Arabia; eoalthubaiti@kau.edu.sa (R.M.A.); amalaih@kau.edu.sa (A.A.M.); 2 Computed Tomography Division, Department of Radiology, King Abdulaziz University Hospital, Jeddah 22252, Saudi Arabia; roudinahedrees@hotmail.com

**Keywords:** computed tomography (CT), clinical-indication-based DRLs, dose optimization

## Abstract

**Background**: Although diagnostic reference levels (DRLs) based on anatomical regions are widely used in computed tomography (CT) imaging, a clinical-indication-based approach provides a more accurate representation of daily practice and protocol variation. This study aimed to establish typical radiation doses for common CT clinical indications among adult patients at King Abdulaziz University Hospital (KAUH) in Saudi Arabia. **Methods**: This retrospective cross-sectional study included 298 adult patients who underwent CT examinations between 2020 and 2025 using two dual-source scanners operating in single- and dual-source modes. Demographic data, acquisition parameters, and radiation dose metrics, including CT dose index (CTDI_vol_) and the dose–length product (DLP), were extracted from scanner consoles. Six clinical indications were analyzed: brain trauma, sinusitis, chest metastases (chest Mets), interstitial lung disease (ILD), abdominopelvic metastases (AbdPel Mets), and hernia. **Results**: Typical median CTDI_vol_ values in mGy were 36.4 for brain trauma, 3.4 for sinusitis, 4.9 for chest Mets, 5.6 for ILD, 7.2 for AbdPel Mets and hernia. Corresponding DLP values in mGy·cm were 654, 50, 173, 188, 344, and 369, respectively. Brain trauma demonstrated the highest radiation exposure, whereas sinusitis CT showed the lowest. Most values were comparable to or lower than international DRLs. **Conclusions**: This study provides the first comprehensive clinical-indication-based DRL data in Saudi Arabia beyond anatomical benchmarks, supporting ongoing dose optimization and future national DRL development.

## 1. Introduction

The use of ionizing radiation from medical imaging procedures has markedly increased globally [1]. Computed tomography (CT) is a major contributor to medical radiation exposure worldwide [2]. The United Nations Scientific Committee on Effects of Atomic Radiation (UNSCEAR) reports that CT imaging has replaced many traditional radiographic and fluoroscopic procedures. Between 2000 and 2018, the number of CT exams has increased by about 80%, constituting 62% of the overall medically related collective effective dose [2]. The basic principles of radiation protection, justification and optimization must be used to ensure patients are protected when undergoing medical imaging exams. Therefore, international bodies such as the International Atomic Energy Agency (IAEA) and the International Commission on Radiological Protection (ICRP) have emphasized the need to establish diagnostic reference levels (DRL) as a method to optimize radiation protection in clinical practice [3,4,5]. While DRLs have been implemented worldwide, many of the reported values were based on anatomical location rather than clinical indication [6,7]. According to the ICRP, setting of a DRL without a relevant clinical indication is of little value, as different clinical indications for an exam may require different image quality and different amounts of radiation. For instance, a large multi-center European study on Clinical Diagnostic Reference Levels (EUCLID) in 2021 collected data on ten common CT indications such as stroke, pulmonary embolism, and appendicitis across 19 hospitals [8]. The study demonstrated that DRL values differed markedly between the clinical sites for the same indication, mainly due to differences in protocols and number of scan phases [8]. Moreover, a recent study found that applying anatomical DRLs, which are focused on the anatomical region being scanned, as a benchmark to specific clinical indications such as head trauma can lead to up to 60% dose misestimation, supporting the use of clinical indication DRLs to improve patient safety in medical imaging [9].

In Saudi Arabia, despite previous studies having been carried out for monitoring CT radiation dose [10,11,12], there is a lack of established clinical-indication-based DRLs (IB-DRLs). To our knowledge, only one recent study [13] was carried out to establish IB-DRLs for adult CT head examinations.

Therefore, the aim of this study was to establish locally based DRLs for adult CT examinations at King Abdulaziz University Hospital (KAUH) based on commonly encountered clinical indications and to compare them with internationally established values. A second aim was to analyze the variation in radiation dose between CT scanners and imaging protocols to provide recommendations for protocol optimization for a particular indication.

## 2. Materials and Methods

### 2.1. Study Design

This retrospective cross-sectional study was conducted in the Radiology Department at KAUH, located in Jeddah, Saudi Arabia. Ethical approval for the study was obtained from the Biomedical Ethics Research Committee of the Faculty of Medicine at King Abdulaziz University.

#### Data Collection

Data were retrospectively collected on the most common clinical indications for adult CT examinations performed between 2020 and 2025 in a CT department, equipped with two dual-source energy Siemens-manufactured CT scanners using single-source mode for (Scanner 1) and either single- or dual-source mode for (Scanner 2) (Table 1). Patient assignment to Scanner 1 or Scanner 2 was based on routine clinical workflow and scanner availability at the time of examination rather than predefined selection criteria. The CT Dose Data Collection Sheet provided by the Saudi Food and Drug Authority (SFDA) was used for documentation.

At least 20 patients were included for each CT indication, in accordance with the recommendations of the ICRP (135). For each examination, data retrieved directly from the scanner console included: patient demographic information (age and gender), technical acquisition parameters (tube voltage (kVp), tube current-time product (mAs), pitch, collimation, slice thickness, and scan length), as well as dosimetric indicators, specifically the volume CT dose index (CTDI_vol_) and the dose–length product (DLP). All examinations meeting the predefined eligibility criteria (i.e., adult CT examinations with complete dose-report data acquired using standard imaging protocols) were selected during data extraction and included in the analysis, with no further exclusions applied thereafter.

### 2.2. CT Equipment and Quality Control

Both CT machines were maintained under a certified quality control (QC) program in accordance with hospital protocols and local regulatory requirements. This included regular, traceable calibration to ensure compliance with dose accuracy standards. Dose metrics (CTDI_vol_ and DLP) were obtained directly from the CT scanner dose reports, which are subject to these routine QA and calibration procedures, ensuring traceability of measurements. While inherent uncertainties in CT dose estimation exist, these are generally within accepted limits for clinical practice. As such, additional verification measurements of the displayed and measured dose values were not necessary.

### 2.3. Data Analysis

The statistical analysis was performed using the IBM SPSS software version 31.0.1.0.

Typical IB-DRLs were determined in accordance with the methodology outlined in ICRP 135 [5]. Typical doses were defined as the median CTDI_vol_ for each scan phase and the cumulative DLP for the entire examination. For each clinical indication, descriptive statistics were used to calculate the median dose values separately for each scanner and for the combined dataset from both CT scanners. Additionally, the 25th and 75th percentiles, minimum, maximum, and standard deviation were estimated to describe central tendency and variability according to the nature of the variables.

The established indication-based typical doses at KAUH were compared with locally and internationally published reference values based on relevance to adult CT examinations and availability of DRL data for comparable clinical indications. Studies with insufficient methodological detail or non-comparable populations were excluded.

## 3. Results

### 3.1. Study Population

The study cohort included 298 adult patients who underwent CT scans for six common clinical indications: brain trauma, sinusitis, chest metastases (chest Mets), interstitial lung disease (ILD), abdominopelvic metastases (AbdPel Mets), and hernia. These examinations were performed using two CT scanners (Scanner 1 and Scanner 2) in our department.

The overall median age of the cohort was 51.6 years ± 8.2, with a female predominance (54.4%). This predominance was most notable in the ILD group (60%), while the other indications showed approximately balanced gender distributions. BMI values predominantly fell within the overweight range (25–30 kg/m^2^), with no notable variation between scanners or clinical indications. The demographic distribution across the six clinical indications, acquired from both scanners, is presented in Table 2.

### 3.2. CT Characteristics

Two dual-source CT scanners were used in this study—Scanner 1 (SOMATOM Definition Flash, installed in 2012) and Scanner 2 (SOMATOM Force, installed in 2024)—both manufactured by Siemens (Table 1). Both scanners were configured with automatic exposure control (AEC) and reported dose metrics, ensuring consistent dosimetric data collection across the study.

### 3.3. CT Scanning Protocols and Technical Parameters

Detailed scanning protocols and technical acquisition parameters for each clinical indication and CT scanner, including mean kVp, mAs, slice thickness, pitch, and scan length, are provided in Table 3. These parameters highlight differences in protocol configurations between scanners and across indications.

Analysis of CT acquisition parameters (Table 3) shows that Scanner 1, operating in single-source mode, generally uses more standardized imaging protocols, with relatively consistent tube voltage (kVp) and tube current-time product (mAs) settings. In contrast, Scanner 2, functioning as a dual-source system, provides greater technical flexibility. Dual-source imaging often employs dual-energy protocols (90/Sn150 kVp) and shows a wider range of mAs values, particularly in abdominopelvic and chest examinations. Across the dataset, the highest exposure values were observed in head trauma examinations in single-source scans (407 ± 69 mAs) and in dual-energy abdominal scans (152 ± 84 mAs), whereas the lowest exposures were recorded in sinusitis CT examinations (38 ± 10 mAs).

Pitch values reflected both standardized protocols and indication-specific variation across the two CT scanners. For brain, sinus, and ILD examinations, pitch remained consistent between both scanners, with fixed values of 0.55 (lowest pitch), 0.8, and 1.2, respectively. In contrast, for abdominopelvic examinations, Scanner 2 consistently utilized a fixed pitch of 0.6 for all source modes. The highest pitch settings were observed in chest imaging, where pitch values were ≥1.2 for both scanners across all examinations.

Slice thickness remains highly consistent across most examinations with 1.5 mm for chest examinations and 2 mm for the head and abdomen scans. Similarly, scan length was determined by the anatomical region being examined, with the greatest coverage observed in hernia imaging (approximately 470 mm) and the shortest in sinus CT studies (approximately 121 mm).

### 3.4. Radiation Dose Comparison Between CT Scanners

The dose metrics, including CTDI_vol_ and DLP, showed variability across clinical indications, scanner types, and acquisition mode (single vs. dual), as shown in Table 4.

Brain trauma examinations produced the highest radiation exposure levels, while sinus examinations showed the lowest doses, with median CTDI_vol_ values ranging from 33.64 to 39.06 mGy and from 3.09 to 3.62 mGy for brain trauma and sinuses, respectively. Between the two units, Scanner 1 consistently delivered higher radiation doses than Scanner 2 operating in single-source mode across all clinical indications. The most pronounced difference was observed in chest Mets imaging, where the median CTDI_vol_ for Scanner 1 (7.12 mGy) was approximately 2.6 times higher than that of Scanner 2 (2.62 mGy). In contrast, sinus imaging was the only clinical indication in which Scanner 2 recorded a slightly higher radiation dose in terms of DLP, with a median value of 55.7 mGy·cm, compared with 45 mGy·cm for Scanner 1.

**Figure 1:**
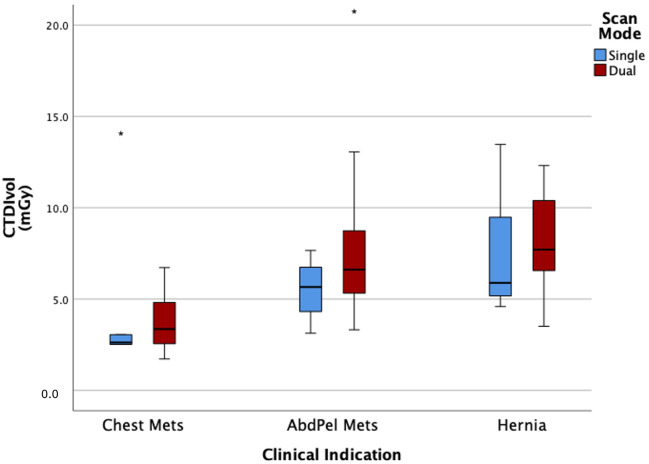
Comparison of CTDI_vol_ in single- and dual-scan modes for Scanner 2 across three clinical indications. The boxplots show the median (central line), interquartile range (IQR; box), min-max values (whiskers) and asterisks (outliers).

**Figure 2:**
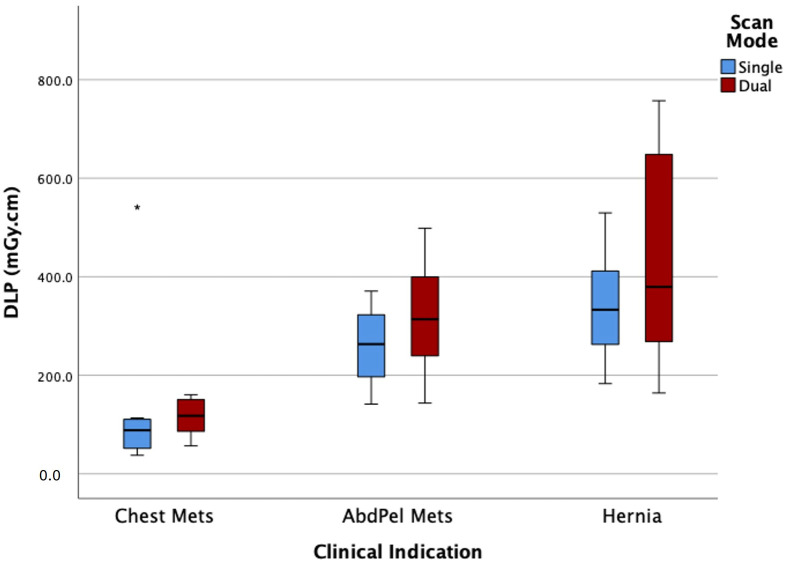
Comparison of DLP in single and dual-scan modes for Scanner 2 across three clinical indications. The boxplots show the median (central line), interquartile range (IQR; box), min-max values (whiskers) and asterisks (outliers).

Regarding the acquisition mode, a slight variation in radiation dose was observed between single-source and dual-source CT acquisitions within the same scanner (Scanner 2) across the examined clinical indications. Overall, dual-source imaging showed marginally higher CTDI_vol_ and DLP values compared to single-source scans, with minimal differences across most indications. For example, AbdPel Mets showed 5.66 mGy and 263 mGy·cm for the single CT acquisition and 6.61 mGy and 313 mGy·cm for the dual CT acquisition (Figure 1 and Figure 2).

Considerable variation was also observed within the same clinical indication. For example, in AbdPel Mets imaging performed on Scanner 1, the DLP increased from a median value of 381.50 mGy·cm to a 75th percentile value of 517 mGy·cm, indicating notable variability within this clinical indication. Such variations were also evident within the same scanner for chest Mets and ILD examinations, reflecting the presence of potential outlier values which likely arises from variability in patient characteristics and scan protocols rather than data inconsistencies.

### 3.5. Establishment of Clinical-Indication-Specific Typical Reference Doses

**Figure 3:**
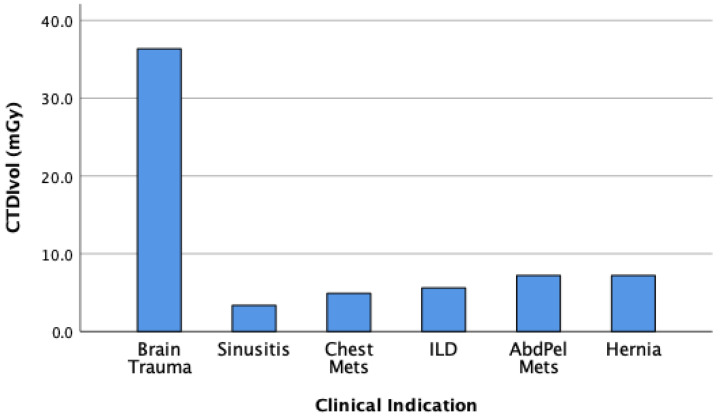
Typical radiation dose (median CTDI_vol_) across clinical indications.

**Figure 4:**
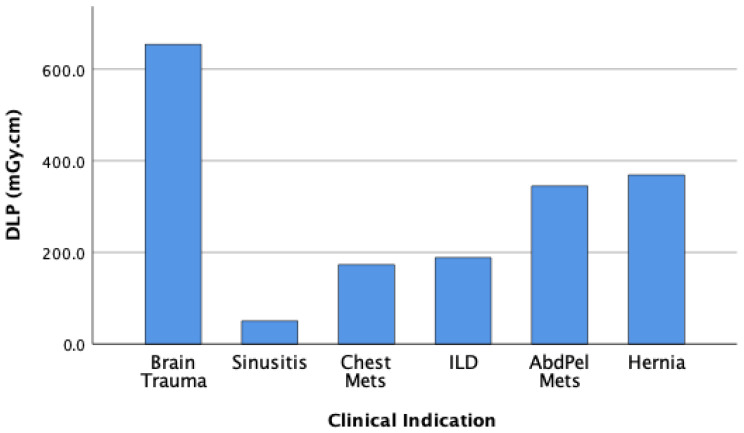
Typical dose–length product (median DLP) across clinical indications.

Figure 3 and Figure 4 present the clinical-indication-based typical doses for the study cohort, expressed as median CTDI_vol_ (mGy) and DLP (mGy·cm). Dose analysis revealed considerable variability across the examined clinical indications. Brain trauma examinations revealed the highest radiation exposure, with a typical reference CTDI_vol_ of 36.4 mGy and a DLP of 654 mGy·cm. In contrast, sinus imaging recorded the lowest radiation levels, with a CTDI_vol_ of 3.4 mGy and a DLP of 50 mGy·cm. Intermediate exposure levels were observed for thoracic protocols, including chest Mets (CTDI_vol_ 4.9 mGy; DLP 173 mGy·cm) and ILD (CTDI_vol_ 5.6 mGy; DLP 188 mGy·cm). For abdominopelvic imaging, both metastatic and hernia evaluations demonstrated comparatively higher radiation doses than thoracic protocols, with a CTDI_vol_ of 7.2 mGy, and corresponding DLP values of 344 mGy·cm and 369 mGy·cm, respectively. These findings were compared to established DRLs reported in international studies and demonstrated overall lower values, as presented in Table 5 and Table 6.

## 4. Discussion

The purpose of our study was to establish the typical doses for common CT clinical indications among adult patients at King Abdulaziz University Hospital in Saudi Arabia. This study represents one of the first in Saudi Arabia to establish DRLs based on specific clinical indications rather than anatomical regions, apart from one previously published study which focused on adult head clinical indications only [13]. Recent international research increasingly supports the use of clinical-indication-based DRLs, as they more accurately reflect real clinical practice and protocol variation across institutions [8,17].

Six common clinical indications were examined including brain trauma, sinuses, chest Mets, ILD, AbdPel Mets and hernia CT scans, performed using two different dual-source CT systems alternating between single- and dual-source modes. Dose variations were noted between the two scanners, among the six clinical indications, within the same clinical indication and between the acquisition modes. These differences were likely attributable to variations in scan mode and imaging protocols, including exposure parameters, essentially mAs, pitch, and slice thickness.

In this study, Scanner 1 operates exclusively in a single-source mode for all routine examinations considered in this study. Scanner 2, by contrast, is utilized in both single- and dual-source modes. Only chest Mets, AbdPel Mets, and hernia protocols were performed in either single or dual-source modes. Across both scanners, head CTs, including common indications such as trauma and sinus evaluation, and ILD were consistently performed using the single-source mode.

The highest radiation dose values were recorded for head trauma examinations across both CT scanners, while the lowest doses were observed in sinus imaging procedures. This is justified by the clinical and technical requirements of head trauma CT scans, which often require tailored exposure settings, including higher mAs, extended scan length, and lower pitch values, all of which contribute to an increased radiation dose. In addition, stricter image quality is required to ensure accurate detection of intracranial hemorrhage or subtle traumatic injuries [21]. Overall, these parameters show no notable differences between scanners suggesting that they are primarily determined by clinical requirements rather than the scanner configuration itself. In contrast, sinus CT examinations are generally confined to a smaller anatomical area and are often performed using dedicated low-dose protocols, given the lower diagnostic complexity and focus on bony structures. As reported by Hoxworth et al., low-dose sinus CT can be performed with a DLP as low as 37.4 mGy·cm without compromising diagnostic accuracy [21]. Meanwhile, studies on trauma head CT indicate significantly higher radiation exposures and wider inter-institutional dose variability due to protocol complexity [22,23].

The comparison of radiation dose between the two CT scanners operating in the same scan mode revealed clear dose variations that were primarily influenced by differences in scanner technology, exposure modulation, and protocol design. Overall, Scanner 2 achieved lower radiation doses for most examinations, particularly in chest Mets imaging which may reflect variability of applied kVp (90 for Scanner 2 and 120 for Scanner 1). Those variations may also be attributable to differences in scanner generation and hardware advancements, as newer CT systems generally offer enhanced dose optimization and improved imaging performance [24,25].

Although dual-source CT is generally associated with dose-efficient imaging and, in some cases, lower radiation exposure compared to single-source systems [24,25], the findings of this study demonstrated slightly higher doses in dual-source mode compared to single-source mode. This discrepancy can be attributed primarily to protocol-specific factors rather than the dual-source technology itself. In the present study, for AbdPel Mets and hernia imaging, the slightly higher radiation dose observed in dual-source mode is likely related to dual-energy acquisition with the use of higher effective mAs values compared to single-source imaging (Table 3), resulting in increased radiation output and a modest rise in dose values. In chest Mets imaging, the slightly higher radiation dose observed in dual-source mode may be attributed to the lower pitch (0.55), which increases beam overlap and dose deposition, despite similar or lower mAs values compared to single-source acquisition. This observation is consistent with previous studies indicating that radiation dose in dual-energy CT is highly dependent on acquisition protocols and may vary depending on the balance between image quality and dose optimization [24,26,27]. Therefore, the slightly higher dose observed in this study likely reflects the impact of applied specific protocols, rather than an inherent limitation of dual-source CT technology. These findings emphasize the importance of ongoing review and optimization of acquisition protocols, particularly in dual-source imaging, to ensure an appropriate balance between image quality and radiation dose.

Not only were dose variations observed between the two scanners and across different clinical indications and scan modes, but they were also evident within the same clinical indication examined by the same scanner. In particular, AbdPel Mets and chest Mets show noticeable outliers and wider dispersion of values, indicating variability in radiation dose among patients undergoing the same examination.

This variability arises because patient-specific factors such as body size (BMI) and anatomical region influence the scanner’s automatic exposure control (AEC) system, which adjusts mAs and kVp in real time to optimize dose and image quality [28,29]. Additionally, differences in clinical requirements, such as the need for higher spatial resolution or enhanced contrast in certain cases, lead to deliberate adjustments in exposure parameters [24]. This difference can also be attributed to variations in individual operator practice, such as protocol adjustments and the selection of scanning parameters during image acquisition.

The comparison of CTDI_vol_ and DLP values with internationally published data indicates that radiation doses in this study were generally lower across most clinical indications (Table 5 and Table 6). For brain trauma, both CTDI_vol_ and DLP were markedly below the values reported from Ghana [14], Norway [15], France [18], and Ireland [20], suggesting reduced scanner output and overall examination dose. A similar pattern was observed for sinusitis, where this study’s CTDI_vol_ and DLP were substantially lower than European surveys [8], EUCLID [17], and Irish benchmarks [20]. For ILD and AbdPel Mets, the doses were also comparable to or lower than the available international reference levels, especially those from Uganda [19]. Although the study population demonstrated relatively higher BMI values, the reported dose levels remained comparable to those in the literature, suggesting that the applied imaging protocols were appropriately optimized and that BMI did not significantly influence inter-scanner comparisons in this study.

This study has several notable strengths. It adopts a clinical-indication-based approach rather than a purely anatomical one, allowing for a more meaningful assessment of dose variation that reflects real-world diagnostic practices and patient-specific imaging needs. This approach provides valuable insight into how CT protocols perform across different clinical contexts, such as trauma, metastasis, and ILD. Moreover, to the best of our knowledge, this is the first study of its kind conducted in Saudi Arabia, establishing a local benchmark for clinical-indication-based DRL for different anatomical regions.

However, the study also has limitations. The findings are limited in generalizability since the data were obtained from a single hospital, where scanner models, operator practices, and protocol settings may differ from those in other institutions. Also, the sample size was relatively modest, which may limit the statistical power to detect subtle differences across all clinical categories. In addition, although formal quantitative image quality assessment was not performed, all included CT examinations followed routine clinical protocols with predefined image quality parameters and were approved by radiologists for diagnostic interpretation. This supports the clinical relevance of the indication-based DRLs; however, the lack of objective image quality evaluation limits direct assessment of the correlation between radiation dose and diagnostic performance. Therefore, while the results offer important baseline information, broader multi-center studies incorporating objective image quality analysis are recommended to validate and extend these observations across different healthcare settings in the region.

## 5. Conclusions

This study established typical radiation doses for six common CT clinical indications among adults at King Abdulaziz University Hospital, using a clinical-indication-based DRL approach. It is the first of its kind in Saudi Arabia to extend beyond anatomical benchmarks. Dose variations were observed between scanners, scan modes and clinical indications as well as within the same clinical indication. These variations were largely influenced by differences in protocols, exposure parameters, and patient-specific factors. When compared with international DRLs, the doses in this study were mostly lower or comparable, indicating effective local dose optimization. These findings provide a reference for dose optimization and a baseline for future national DRL development, potentially supporting protocol standardization efforts across healthcare facilities in Saudi Arabia.

## Figures and Tables

**Table 1:** Technical characteristics of the CT scanners used in the study.

CT Scanner Specification	Scanner 1	Scanner 2
CT manufacturer	SIEMENS	SIEMENS
CT model	SOMATOM Definition Flash	SOMATOM Force
Year of installation	2012	2024
Detector type	2 × Stellar detector	2 × Stellar Infinity detectors with anti-scatter 3D collimator grid
Number of detector rows	2 × 128	2 × 192
Number of slices acquired simultaneously	2 × 128 (acquired slices)	2 × 192 (acquired slices)
Reconstruction slice width option	0.5–10 mm	0.4–20 mm
Automatic exposure control	YES	YES
* CTDI_w_/CTDI_vol_	CTDI_vol_	CTDI_vol_

* CTDI_w_: weighted CT dose index, CTDI_vol_: volume CT dose index.

**Table 2:** Distribution of patient characteristics by CT scanner type.

Indication	CT Scanner Type	Total No of Patients (298)	Gender *N* (%)		Age (y) Mean ± SD	Weight (kg) Mean ± SD	Height (cm) Mean ± SD	BMI (kg/m^2^) Mean ± SD
			Male 136 (45.6)	Female 162 (54.4)				
Brain Trauma	Scanner 1	24	9 (37.5)	15 (62.5)	47 ± 17	69 ± 19	161 ± 10	27 ± 9
	Scanner 2	23	10 (43.5)	13 (56.5)	46 ± 22	65 ± 15	161 ± 9	25 ± 6
Sinusitis	Scanner 1	25	12 (48.0)	13 (52.0)	40 ± 12	72 ± 16	162 ± 11	28 ± 7
	Scanner 2	26	11 (42.3)	15 (57.7)	36 ± 9	69 ± 18	163 ± 6	26 ± 6
Chest Mets	Scanner 1	25	13 (52.0)	12 (48.0)	58 ± 13	76 ± 11	163 ± 5	28 ± 3
	Scanner 2	25	12 (48.0)	13 (52.0)	60 ± 17	65 ± 10	160 ± 10	25 ± 4
ILD	Scanner 1	24	9 (37.5)	15 (62.5)	55 ± 19	71 ± 23	158 ± 10	29 ± 8
	Scanner 2	25	10 (38.5)	15 (61.5)	55 ± 18	70 ± 17	159 ± 8	28 ± 7
AbdPel Mets	Scanner 1	24	12 (50.0)	12 (50.0)	56 ± 14	74 ± 13	162 ± 10	28 ± 4
	Scanner 2	26	13 (50.0)	13 (50.0)	62 ± 17	68 ± 10	163 ± 11	26 ± 5
Hernia	Scanner 1	23	11 (47.8)	12 (52.2)	51 ± 13	76 ± 12	160 ± 7	30 ± 6
	Scanner 2	28	14 (50.0)	14 (50.0)	53 ± 16	75 ± 15	159 ± 9	30 ± 7

Mets: metastasis, ILD: interstitial lung disease, AbdPel: abdominopelvic.

**Table 3:** Comparison of CT imaging parameters (mean ± SD) for Scanner 1 and Scanner 2 across clinical indications.

Indication	CT Scanner	Scan Mode	Tube Voltage (kVp, Mode)	Tube Current-Time Product (mAs)		Pitch	Slice Thickness (mm)	Scan Length (cm)
				Effective Mean ± SD	Reference Mean ± SD			Mean ± SD
Brain Trauma	Scanner 1	Single source	100	407 ± 69	561 ± 49	0.55	2	165.5 ± 9.6
	Scanner 2	Single source	120	224 ± 24	280 ± 0	0.55	2	165.7 ± 11.1
Sinusitis	Scanner 1	Single source	100	38 ± 10	47 ± 10	0.80	2	121.6 ± 9.8
	Scanner 2	Single source	100	45 ± 14	46 ± 0.2	0.80	2	120.8 ± 14.8
Chest Mets	Scanner 1	Single source	120	108 ± 37	96 ± 7.5	1.2	1.5	304.7 ± 36.7
	Scanner 2	Single source	90	81 ± 55	88 ± 46	1.2/3	1.5	314.3 ± 21.6
		Dual source	90/Sn150	Tube A: 80 ± 45 Tube B: 52 ± 22	Tube A: 60 ± 0 Tube B: 46 ± 0	0.55	1.5	292.3 ± 40.3
ILD	Scanner 1	Single source	120	129 ± 43	119 ± 30	1.2	1.5	290.9 ± 42.1
	Scanner 2	Single source	110	76 ± 26	74 ± 11	1.2	1.5	289.5 ± 38.4
AbdPel Mets	Scanner 1	Single source	120	146 ± 53	158 ± 23	1	2	442.4 ± 32.6
	Scanner 2	Single source	90	128 ± 24	205 ± 48	1	2	389.0 ± 108.9
		Dual source	90/Sn150	Tube A: 152 ± 84 Tube B: 90 ± 36	Tube A: 175 ± 0 Tube B: 109 ± 0	0.6	2	442.7 ± 42.0
Hernia	Scanner 1	Single source	120	131 ± 28	160 ± 23	0.6/0.85/1	2	450.8 ± 39.3
	Scanner 2	Single source	110	125 ± 38	173 ± 52	0.6	2	472.5 ± 75.2
		Dual source	90/Sn150	Tube A: 166 ± 70 Tube B: 93 ± 30	Tube A: 144 ± 35 Tube B: 90 ± 21	0.6	2	477.0 ± 97.8

Mets: metastases, ILD: interstitial lung disease, AbdPel: abdominopelvic. kVp = peak tube potential applied during CT acquisition. Values shown represent the most frequently used tube voltage setting (mode) for each clinical indication. Pitch and slice thickness represent fixed settings applied consistently across examinations within each clinical indication.

**Table 4:** Summary of CTDI_vol_, DLP across clinical indications and scanner types.

		CTDI_vol_ (mGy)				DLP (mGy·cm)			
Indication	Scanner	Mean ± SD	25th Percentile	50th Percentile	75th Percentile	Mean ± SD	25th Percentile	50th Percentile	75th Percentile
Brain Trauma	Scanner 1	39.82 ± 5.8	35.7	39.1	42.6	726.4 ± 145.4	643.0	692.0	761.3
	Scanner 2	34.00 ± 3.7	31.6	33.6	35.8	622.7 ± 80.0	555.5	616.3	678.5
Sinusitis	Scanner 1	3.29 ± 0.8	2.9	3.1	3.4	47.8 ± 12.8	41.0	45.0	51.0
	Scanner 2	4.00 ± 1.2	3.2	3.6	4.1	61.1 ± 20.1	47.6	55.7	67.0
Chest Mets	Scanner 1	7.69 ± 3.3	5.9	7.1	8.6	256.7 ± 103.9	189.0	243.0	304.5
	Scanner 2	3.79 ± 2.8	2.4	2.7	4.7	131.1 ± 103.7	82.2	103.3	150.3
ILD	Scanner 1	7.58 ± 2.6	5.2	7.2	9.6	250.8 ± 91.6	175.8	246.5	307.0
	Scanner 2	4.19 ± 2.0	2.7	4.1	5.0	141.9 ± 62.3	98.6	130.0	176.4
AbdPel Mets	Scanner 1	9.42 ± 3.9	6.7	8.3	10.8	442.6 ± 188.3	316.8	381.5	517.0
	Scanner 2	7.20 ± 3.5	5.2	6.1	8.6	344.2 ± 180.3	238.6	308.0	397.8
Hernia	Scanner 1	8.87 ± 3.1	7.5	8.3	9.9	423.8 ± 141.6	355.0	405.0	448.0
	Scanner 2	7.22 ± 2.7	5.2	6.1	9.6	368.6 ± 147.3	264.0	333.1	448.2

CTDI_vol_: volume CT dose index, DLP: dose–length product, DRL: diagnostic reference levels, Chest Mets: chest metastases, ILD: interstitial lung disease, AbdPel Mets: abdominopelvic metastases.

**Table 5:** Comparison of the overall clinical-indication-based CTDI_vol_ (mGy) of this study with international values.

Study	CTDI_vol_ (mGy)					
	Brain Trauma	Sinusitis	Chest Mets	ILD	AbdPel Mets	Hernia
This study	36.4	3.4	4.9	5.6	7.2	7.2
Ghana [14]	77.0	-	-	-	17.0	-
Norway [15]	60.0	-	-	-	11.0	-
Switzerland [16]	-	-	-	-	11.0	-
European survey [8]	-	11.0	-		-	-
EUCLID [17]	-	21.0	-		-	-
France [18]	43.0	-	-	-	12.0	-
Uganda [19]	32.0	-	-	4.7	-	-
Ireland [20]	63.0	21.0	-	7.0	-	-

CTDI_vol_: volume CT dose index, Chest Mets: chest metastases, ILD: interstitial lung disease, AbdPel Mets: abdominopelvic metastases.

**Table 6:** Comparison of the overall clinical-indication-based DLP (mGy·cm) of this study with international values.

Study	DLP (mGy·cm)					
	Brain Trauma	Sinusitis	Chest Mets	ILD	AbdPel Mets	Hernia
This study	654.2	50.4	173.2	188.3	344.7	369.1
Ghana [14]	1596.0	-	-	-	1299.0	-
Norway [15]	950.0	-	-	-	800.0	-
Switzerland [16]	-	-	-	-	540.0	-
European survey [8]	-	181.0	-	-	-	-
EUCLID [17]	-	373.0	-	-	-	-
France [18]	920.0	-	-	-	880.0	-
Uganda [19]	878.0	-	-	161.0	-	-
Ireland [20]	922.5	196.5	-	229.5	-	-

DLP: dose–length product, Chest Mets: chest metastases, ILD: interstitial lung disease, AbdPel Mets: abdominopelvic metastases.

## Data Availability

The data presented in this study are available upon request from the corresponding author due to ethical and privacy restrictions related to patient data.

## Abbreviations

The following abbreviations are used in this manuscript:

AbdPel Mets	Abdominopelvic metastases
AEC	Automatic exposure control
Chest Mets	Chest metastases
CTDI_vol_	Volume CT dose index
CTDI_w_	Weighted CT dose index
CT	Computed tomography
DLP	Dose–length product
DRLs	Diagnostic reference levels
EUCLID	European Study on Clinical Diagnostic Reference Levels
IAEA	International Atomic Energy Agency
ICRP	International Commission on Radiological Protection
ILD	Interstitial lung disease
kVp	Tube voltage
mAs	Tube current-time product
QC	Quality control
SFDA	Saudi Food and Drug Authority
UNSCEAR	United Nations Scientific Committee on Effects of Atomic Radiation
